# Multimodal Affective State Assessment Using fNIRS + EEG and Spontaneous Facial Expression

**DOI:** 10.3390/brainsci10020085

**Published:** 2020-02-06

**Authors:** Yanjia Sun, Hasan Ayaz, Ali N. Akansu

**Affiliations:** 1 Department of Electrical and Computer Engineering, New Jersey Institute of Technology, Newark, NJ 07102, USA; akansu@njit.edu; 2 School of Biomedical Engineering, Science and Health Systems, Drexel University, Philadelphia, PA 19104, USA; hasan.ayaz@drexel.edu; 3 Department of Psychology, College of Arts and Sciences, Drexel University, Philadelphia, PA 19104, USA; 4 Department of Family and Community Health, University of Pennsylvania, Philadelphia, PA 19104, USA; 5 Center for Injury Research and Prevention, Children’s Hospital of Philadelphia, Philadelphia, PA 19104, USA

**Keywords:** functional near-infrared spectroscopy (fNIRS), electroencephalography (EEG), facial emotion recognition, brain–computer interface (BCI)

## Abstract

Human facial expressions are regarded as a vital indicator of one’s emotion and intention, and even reveal the state of health and wellbeing. Emotional states have been associated with information processing within and between subcortical and cortical areas of the brain, including the amygdala and prefrontal cortex. In this study, we evaluated the relationship between spontaneous human facial affective expressions and multi-modal brain activity measured via non-invasive and wearable sensors: functional near-infrared spectroscopy (fNIRS) and electroencephalography (EEG) signals. The affective states of twelve male participants detected via fNIRS, EEG, and spontaneous facial expressions were investigated in response to both image-content stimuli and video-content stimuli. We propose a method to jointly evaluate fNIRS and EEG signals for affective state detection (emotional valence as positive or negative). Experimental results reveal a strong correlation between spontaneous facial affective expressions and the perceived emotional valence. Moreover, the affective states were estimated by the fNIRS, EEG, and fNIRS + EEG brain activity measurements. We show that the proposed EEG + fNIRS hybrid method outperforms fNIRS-only and EEG-only approaches. Our findings indicate that the dynamic (video-content based) stimuli triggers a larger affective response than the static (image-content based) stimuli. These findings also suggest joint utilization of facial expression and wearable neuroimaging, fNIRS, and EEG, for improved emotional analysis and affective brain–computer interface applications.

## 1. Introduction

The face has long been considered as a window with a view to our emotions [1]. Facial expressions are regarded as one of the most natural and efficient cues enabling people to interact and communicate with others in a nonverbal manner [2]. With the systematic analysis of facial expression [3], the link between facial expression and emotion has been demonstrated empirically in psychology literature [1,4]. Decades of behavioral research revealed that facial expression carries information for a wide-range of phenomena, from psychopathology to consumer preferences [5,6,7]. The recent advances in electronics and computational technologies allow recording facial expressions at increasingly high resolutions and advanced the analysis performance. A better understanding of facial expressions can contribute to human-computer interactions and emerging practical applications that employ facial expression recognition, such as in education, entertainment, interactive games, clinical diagnostics, and many others.

When and how to capture spontaneous facial expressions, as well as the methods to interpret associated mental states and the underlying neurological mechanisms are growing research areas [8,9,10]. In this study, we extended our previous work [11] to investigate the relationship between spontaneous human facial emotion analysis and brain signals generated due to reactions to both static (image) and dynamic (video) stimuli. We jointly analyze the affective states by using multimodal brain activity measurements. The facial emotion recognition method utilizes image processing and pattern recognition and classification to decode the universal emotion types [12]. Namely, these primitive emotions are anger, disgust, fear, happiness, sadness, and surprise [13]. Facial expressions can be coded by the facial action coding system (FACS) which describes an expression through the action units (AU) of individual muscles [14]. Although facial expression descriptions may be precise, automatic recognition of the emotions behind specific facial expressions from images remains a challenge without the availability of context information [10]. Some existing image classification methods have achieved high recognition rates for facial emotions based on benchmarked databases containing a variety of posed facial emotions [15,16]. However, these datasets are built from images of subjects performing exaggerated expressions that are quite different than spontaneous and natural presentations [17].

The neural mechanisms of emotion processing have been a fundamental research area in cognitive neuroscience and psychiatry in part due to clinical applications relating to mood disorders [18,19]. Researchers have shown that neurophysiological changes are induced by non-consciously perceived emotional stimuli [20]. In particular, prefrontal cortex (PFC) has been identified as an important region that facilitates emotion regulation and, as a result, functional neuroimaging of PFC has been used to investigate neural correlates of emotion processing [21,22,23,24,25]. Findings from these studies have suggested that monitoring PFC activity using non-invasive neuroimaging approaches, including functional near-infrared spectroscopy (fNIRS) [26] and electroencephalography (EEG) [27], presents an opportunity for automatic emotion recognition. These tools enable measuring the brain activity in natural everyday settings with minimal restrictions on participants during measurement. Hence, they are ideal tools for the Neuroergonomics approach [28,29,30] that is focusing on studying the brain with real/realistic settings as opposed to artificial lab settings. Findings from these tools can be used for mapping the brain function as well as decoding mental states.

fNIRS is a non-invasive and portable neuroimaging method that can quantify the changes of cerebral oxygenated and deoxygenated hemoglobin concentrations using near-infrared light attenuation. fNIRS measures cortical hemodynamic response similarly to functional magnetic resonance imaging (fMRI), but without limitations and restrictions on the subject such as staying in a supine position within a confined space or exposure to loud noises [31]. As a portable and cost-effective functional neuroimaging modality, fNIRS is uniquely suitable to study cognition and emotion processing-related brain activities due to relatively high spatial resolution and a practical sensory setup [22,31,32,33,34]. EEG is a non-invasive, portable, and widely adopted neuroimaging technique used to detect brain electrophysiological patterns. It measures electrical potentials through electrodes placed on the scalp. Due to its high temporal resolution, EEG is an ideal candidate for monitoring event-related brain dynamics. Furthermore, EEG has been widely used to investigate the brain signals implicated in emotion processing [35,36]. It has been reported that asymmetric brain activity in frontal region is a key biomarker observed for emotional stimuli using EEG, fNIRS and fMRI [37,38,39,40]. Davidson et al. proposed that activity differences between the left and right PFC hemisphere as acquired by EEG were associated with the processing of positive and negative affects [41]. According to this view of frontal asymmetry, the left prefrontal cortex is thought to be associated with positive affect, and the right prefrontal cortex activity is related to negative affect [42].

**Figure 1:**
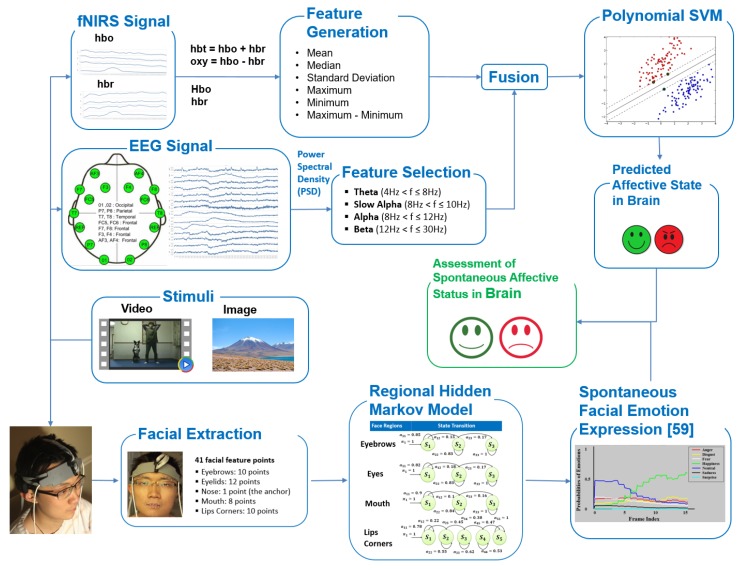
Framework of assessing the spontaneous affective status in brain through comparing brain activity and spontaneous facial emotions. fNIRS: functional near infrared spectroscopy; SVM: Support Vector Machine; EEG: electroencephalography.

The measurement of neural correlates of cognitive and affective processes using concurrent EEG and fNIRS, multimodal functional neuroimaging, has seen growing interest [43,44,45,46]. As fNIRS and EEG measure complementary aspects of brain activity (hemodynamic and electrophysiological, respectively), a hybrid brain data incorporates more information and enabling higher mental decoding accuracy [43] confirming earlier findings [47]. Specifically, in [43] we showed that body physiological measures (heart rate and breathing) did not contribute any new information to fNIRS + EEG based classification of cognitive workload. Another recent study reported in [48] utilized fNIRS and EEG as well as with autonomic nervous system measures, including skin conductance responses and heart rate, for emotion analysis. Authors reported strong effects observed in fNIRS and EEG when comparing positive and negative valence. And, they confirmed prefrontal lateralization for valence. Finally, heart rate didn’t show any effect, but skin conductance response demonstrated a difference although no comparison was done if this adds to EEG or fNIRS. In a more recent study, authors used prefrontal cortex based fNIRS signals recording during emotional video clips to recognize different positive emotions [49]. In this study, we investigated spontaneous facial affective expressions and brain activity simultaneously recorded using both fNIRS and EEG modalities for affective state estimation. The block diagram of the system is displayed in Figure 1.

This paper highlights the benefits of multimodal wearable neuroimaging using ultra-portable battery-operated and wireless sensors that allows for the untethered measurement of participants, ad potentially can be used in everyday settings. The major contributions of the paper are summarized as follows:

- To the best of our knowledge, this is the first attempt to explore the relationship between spontaneous human facial affective states and relevant brain activity by simultaneously using fNIRS, EEG, and facial expressions registered in captured video.
- The spontaneous facial affective expressions recorded by a video camera are demonstrated to be in line with the affective states coded by brain activities. This is consistent with Neuroergonomics [30] and mobile brain/body imaging approaches [50].
- The experimental results show that the proposed multimodal technique outperforms methods using a subset of these signal types for the same task.

The remainder of the paper is organized as follows. Section 2 details the approach and methods as well as the experimental design used in the study. Section 3 reviews analytical details and presents the results. Then, the discussion and concluding remarks are given in the last section of the paper.

## 2. Materials and Methods

### 2.1. Participants

Twelve male participants (age: µ = 27.58, σ = 4.81) volunteered for the study. Each participant gave written informed consent prior to participation in this study. We have opted to recruit only male participants in this study in order to eliminate the confounding factor of menstrual cycle phases’ impact on emotion processing in female volunteers [51,52,53,54]. Participants all self-identified as right-handed and self-reported to have no history of mental illnesses or drug abuse, and were compensated for their time. The study was conducted in accordance with the Declaration of Helsinki and approved by the Institutional Review Board of the New Jersey Institute of Technology.

### 2.2. Experimental Protocol

**Figure 2:**
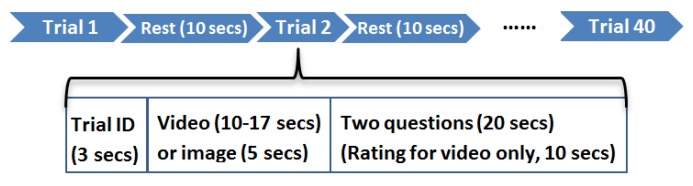
Experimental protocol.

Each participant was assigned to complete two tasks according to the experimental protocol shown in Figure 2. In the first task, each participant was asked to watch twenty videos with various emotional content. Each video lasted 10–17 s such that the participant can recognize the type of affect. After watching a video, the participant answered two simple questions (e.g., Were you able to watch the video carefully? What were you seeing?) in order to verify he understood the video content. In addition, each participant was asked to evaluate the type of affect (positive or negative) and the degree of the affect using a ten-point Likert scale (ranging from 1 = extremely negative to 10 = extremely positive) in response to a set of affective states. It is worth noting that the participant did not know the video contents in advance. The advantage of this experimental procedure is that each participant naturally gives the final ratings in the absence of prior knowledge. In the second task, each participant was asked to observe twenty emotional images from Nencki Affective Picture System (NAPS) [55]. Each image was displayed for five seconds. Analogously, the participant answered two simple questions about the image content after observing the image.

**Figure 3:**
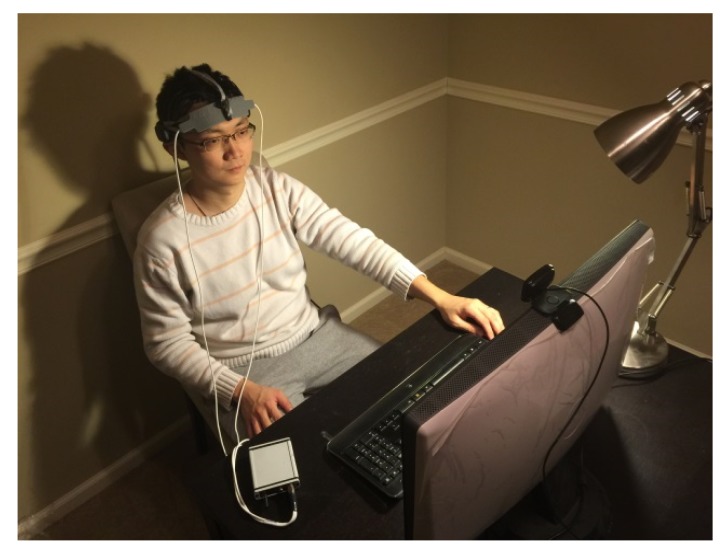
Experimental environment and participant wearing brain monitoring sensors.

Each participant was instructed in the experimental procedure in detail before performing the experiment. Participants were asked to sit on a comfortable chair facing a computer screen in a quiet room. Both fNIRS and EEG sensors were placed on the participant’s forehead and scalp, respectively. There was no contact between these two hardware pieces. During the experiment, participants facial reactions to the stimuli were video recorded by a webcam. Each participant was required to minimize his head movements during the experiment in order to avoid signal artifacts from head movement. The experimental environment is shown in Figure 3. The study has been approved by the Institutional Review Broad of New Jersey Institute of Technology. Before the experiment, each participant was asked to sign an agreement to participate in the study.

### 2.3. Brain Data Acquisition

**Figure 4:**
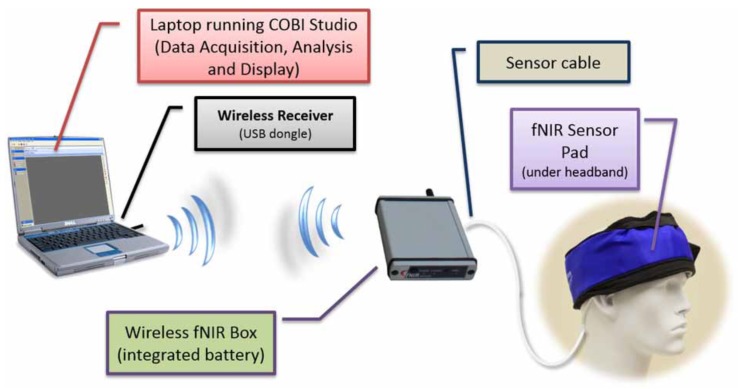
Components of functional near-infrared spectroscopy (fNIRS) system: wireless transmitter, wireless box containing battery, and sensor pad [31].

The neuroimaging systems used in this study consisted of two commercial products: a wireless fNIRS Model 1100W system (www.fnirdevices.com) and an Emotiv EPOC headset (www.emotiv.com). A compact and battery-operated wireless mini-fNIRS system was used to monitor the prefrontal cortex of the participant as shown in Figure 4. The system measures cortical oxygenation changes during the task and is composed of three modules: a sensor pad that holds near-infrared light sources and detectors to enable a fast placement of 4 optodes (2 light wavelengths channels and an ambient channel per optode), control box hardware for sampling all channels at 4 Hz, and a computer that runs COBI Studio software [56] that controls data collection and receives the data wirelessly from the hardware. More information about the device and data collection procedures was reported in [31].

**Figure 5:**
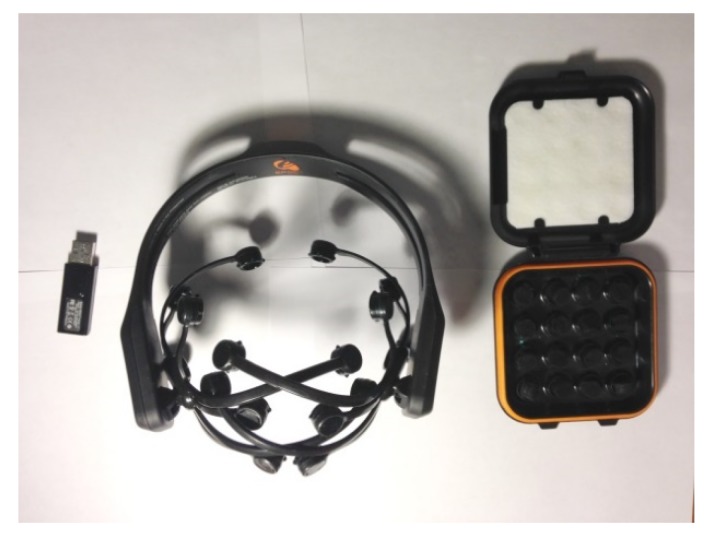
Emotiv EPOC headset: USB Dongle, EPOC headset, and electrodes [57].

The Emotiv EPOC headset shown in Figure 5 acquired 128 Hz EEG signals by measuring electrical differences on the scalp, and then transmitted the signals wirelessly to a Windows PC. The system measures the electrical potentials of the scalp caused by neurons firing. The cap has 14 electrodes located over 10–20 system positions AF3, F7, F3, FC5, T7, P7, O1, O2, P8, T8, FC6, F4, F8, and AF4 using 2 reference electrodes. The saline-soaked felt pad is used to reduce electrical resistance between the skin and the electrodes. Low electrode impedances are achieved using saline solution as indicated by software.

### 2.4. Automatic Facial Emotion Recognition System

**Figure 6:**
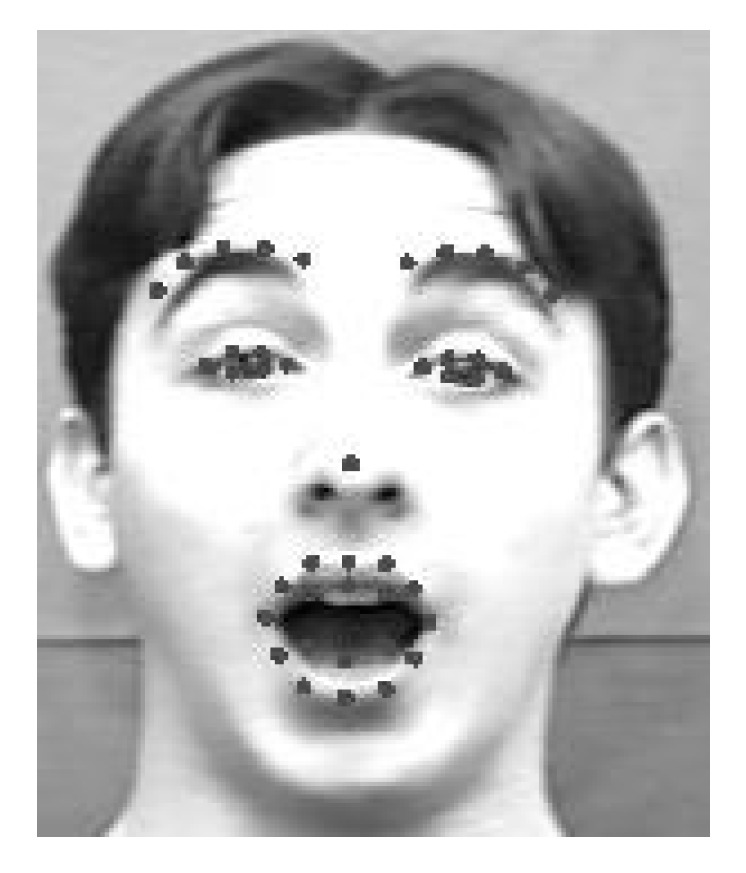
Facial feature points on a face image.

**Figure 7:**
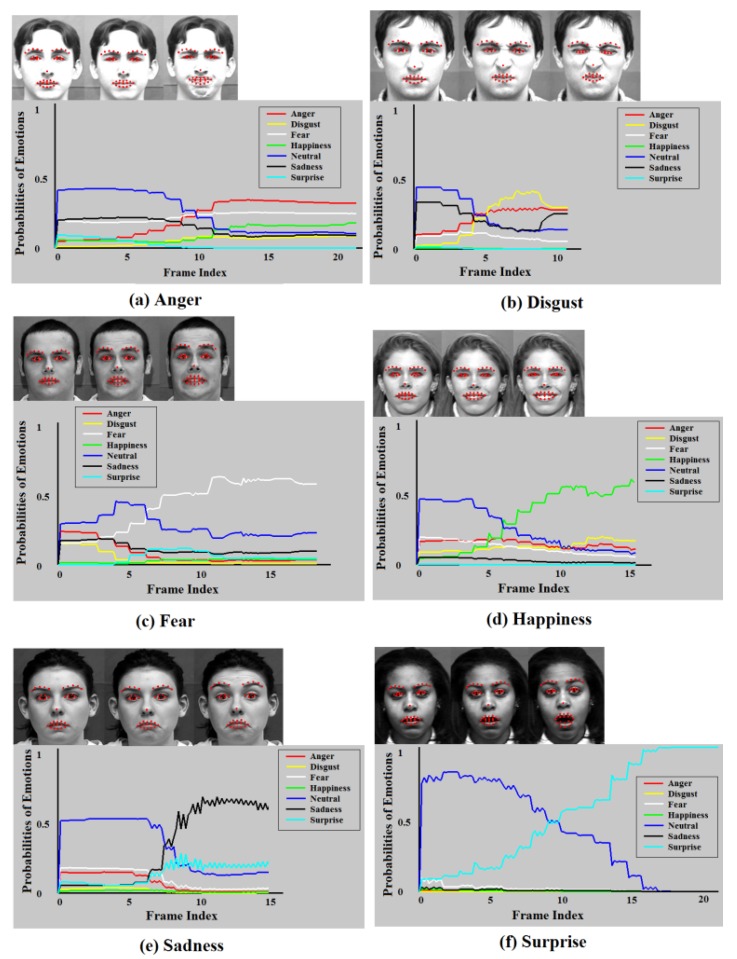
Recognition rates of emotion types as a function of frame index (time) in a video sequence. (**a**) Anger; (**b**) Disgust; (**c**) Fear; (**d**) Happiness; (**e**) Sadness; (**f**) Surprise.

The automatic facial emotion recognition system proposed in [58] is used to identify the spontaneous facial affective states. The system not only outperforms the state-of-the-art based on the posed expressions but also provides satisfactory performance on spontaneous facial emotions. The system has been used to read Chief Executive Officers’ (CEO) facial expressions to forecast firm performance by only using recorded video signal [59]. It utilizes Regional Hidden Markov Model (RHMM) as its classifier to train the states of three face regions: the eyebrows, eyes, and mouth, as tabulated in Table 1. Since the biological information that describes a facial expression is mainly registered in the movement of these three regions as sensed and quantified in frames of a video sequence, it is natural to classify the states of each facial region rather than modeling the entire face. Note from the table that the mouth region is slightly different from the eyebrows and eye regions. In addition to the mouth itself, the lips corners are also important features. Considering a practical application, this system can classify frames as they come into analysis. To describe the states of face regions, 41 facial feature points are identified on each frame of video, as displayed in Figure 6. They are comprised of 10 feature points on the eyebrows region, 12 points on the eyelids, 8 points on the mouth, 10 points on the corners of the lips, and one anchor feature point on the nose. The 2D coordinates of facial feature points in various face regions are extracted to form corresponding observation sequences for classification. As an example, Figure 7 displays the recognition rates for emotion types in each frame as a function of frame index (time) in a video sequence.

The system serves the needs of this study for three main reasons. First, the objective and measurable response to a person’s emotions by the system is perceived as more natural, persuasive, and trustworthy. This allows us to plausibly analyze the measured data. Second, the system can recognize the various affective states of interest. Its last and most important advantage is that the system automatically analyzes and measures the live facial video data in a manner that is intuitive and useful for different applications. It helps the user to take actions based on this analysis.

### 2.5. Stimuli Evaluation

The twenty emotional images used in the trials were obtained from the Nencki Affective Picture System (NAPS) [55]. It is a standardized, wide-range, high-quality, realistic picture database that is widely used for brain imaging studies. We selected ten positive-content images and ten negative-content ones according to the given valence values [11]. Each image is classified explicitly with the attached emotion type if over 50% of all participants express the same facial affect. Otherwise, it is classified as an ambiguous image that is discarded [58]. Eventually, all images are classified explicitly. Therefore, all of them are utilized for the experiments.

For the video-content part of the experiment, we selected twenty videos in English from Youtube.com. They were evenly selected based on the contents (positive and negative) and length (10–17 s). The positive content contains the funny or happy clips, e.g., dogs mimic human’s behaviors like doing exercises, sitting and eating food, pushing the baby stroller, etc. The negative clips show sadness, anger, or disgust, e.g., the people living in poverty, wars, memorial service, etc. The selected videos were independently watched and evaluated by all twelve participants. To make sure the video contents are consistent with participants’ spontaneous affective states, we did the similar initial evaluation as an image assessment to classify all video clips as explicit or ambiguous video. Each video is classified explicitly with the attached emotion type if over 50% of all participants express the same facial affect. Otherwise, it is classified as an ambiguous video that is discarded. Eventually, all selected video clips were classified explicitly and used in the following experiments.

### 2.6. Data Pre-Processing for Brain Activity and Facial Expression

EEG signals were passed through a low-pass filter with 30 Hz cutoff frequency. ICA analysis was performed in EEGLAB, an open-source toolbox for analysis of single-trial EEG dynamics [60]. It was used to detect and remove the artifacts in the raw EEG signals following the approach described in [61]. The average of the 5-s baseline brain signal before each trial was subtracted from the brain response data for baseline adjustment. To capture the affective states (positive or negative) in different brain regions, four frequency bands as Power Spectral Density (PSD) features were extracted from EEG signals to identify brain patterns. The correlation between EEG spectral density in these frequency bands and the spontaneous affective state were compared via 14 electrodes. Additional information about correlations is included in the Appendix A.

A low-pass filter with 0.1 Hz cutoff frequency was used to achieve noise reduction in fNIRS signals [26]. Motion artifacts were eliminated prior to extracting the features from fNIRS signals by applying a fast Independent Component Analysis (ICA) [62]. The independent component was selected through modeling the hemodynamic response. The modeled hemodynamic response represented the expected hemodynamic response to the given stimulus calculated by convolving the stimulus function and a canonical hemodynamic response function (HRF). The HRF [63] consists of a linear combination of two Gamma functions as

(1) $$h\left(t\right)=A(\frac{t^{\alpha_{1}-1}\beta_{1}{}^{\alpha_{1}}e^{-\beta_{1}t}}{\Gamma \left(\alpha_{1}\right)}-c\frac{t^{\alpha_{2}-1}\beta_{2}{}^{\alpha_{2}}e^{-\beta_{2}t}}{\Gamma \left(\alpha_{2}\right)})$$

where A controls the amplitude, α and β control the shape and scale respectively, and c determines the ratio of the response to undershoot. A *t*-test was used to select the independent component associated with the hemodynamic response [64]. It is expected that the independent component with the highest t-value is associated with the hemodynamic response to a given stimulus.

Inference of participants’ facial affective expressions is based on facial emotion recognition. Facial affective expressions can be influenced by various factors including age, gender, race, time of day, and the general health of the participant. To control for these factors, we calibrated our measurements over the first 5 s of facial expressions. During this 5 s calibration period, we computed the mean measures of all emotion states. We then subtracted these mean measures from the remainder of facial expressions for baseline adjustment [59]. The emotion type of a facial video clip v was recognized by calculating the largest probability among Anger, Disgust, Fear, Happiness, Neutral, Sadness, and Surprise expressed as

(2) $$P_{v}{}^{Emotion}=\max\left(P_{i}\right),\;v=1,\;\ldots ,\;N_{video},\;i=1,\;\ldots \;7$$

where $N_{video}$ is the total number of videos clips, each of which contains the participant’s facial response to the stimulus. $P_{i}$ is the probability of an emotion type that is obtained by summing the overall probabilities of this emotion type in each frame of a video clip.

**Figure 8:**
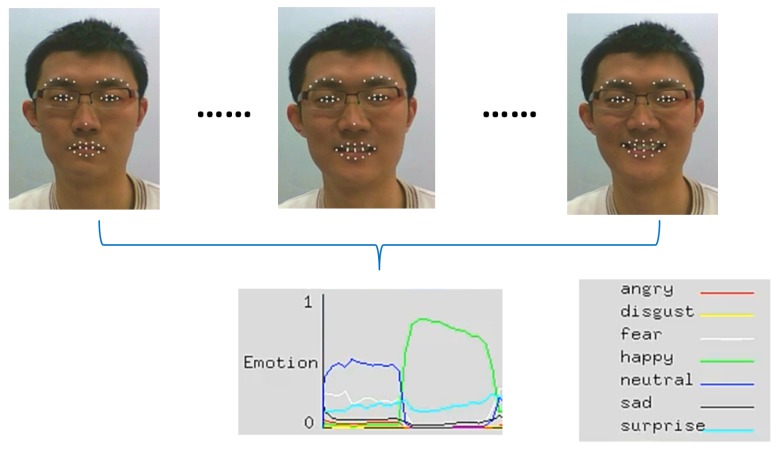
Recognition rates of emotion types as a function of frame index (time) in a video sequence.

Happiness is coded as a positive affect and Anger, Disgust, Fear, and Sadness are regarded as a negative affect. Since Surprise can be revealed as a result of positive or negative affect, it was not used in this experiment. Neutral emotion is neither positive or negative affect, so it was not used in the experiment, either. The ratings of both the positive and the negative affects for a video clip are separately calculated by the system as

(3) $$\begin{array}{c} P_{v}{}^{Affect}=\max\left(P_{pos},\;P_{neg}\right),\;v=1,\;\ldots ,\;N_{video},\\ \mathrm{subject}\;\mathrm{to}\;\{\begin{array}{l} P_{pos}=\frac{P_{Happiness}}{P_{Happiness}+\max\left(P_{Anger},\;P_{Disgust},\;P_{Fear},\;P_{Sadness}\right)}\\ P_{neg}=\frac{\text{max}\left(P_{Anger},\;P_{Disgust},\;P_{Fear},\;P_{Sadness}\right)}{P_{Happiness}+\max(P_{Anger},\;P_{Disgust},\;P_{Fear},\;P_{Sadness})} \end{array} \end{array}$$

where $P_{v}{}^{Affect}$ is the recognized affective state of the corresponding video clip. Figure 8 shows the spontaneous facial affective states of a participant that are detected by system when he is watching videos or images during experiments.

### 2.7. Feature Extraction

The recorded data of raw fNIRS light intensity at two wavelengths (730 nm and 850 nm) is converted to the relative changes in hemodynamic responses in terms of oxy-hemoglobin (Hbo) and deoxy-hemoglobin (Hbr) using the modified Beer-Lambert Law [56]. Total hemoglobin concentration changes (Hbt), the sum of Hbo and Hbr and an estimate of the total blood volume, and the difference of Hbo and Hbr, the estimate of oxygenation change, were also calculated for each optode. We calculated the mean, median, standard deviation, maximum, minimum, and the range of maximum and minimum of four hemodynamic response signals as features, 4 × 4 × 6 = 96 fNIRS features for each trial.

The spectral power of EEG signals in different bands has been used for emotion analysis [65]. The logarithms of the power spectral density (PSD) for theta (4 Hz < f ≤ 8 Hz), slow alpha (8 Hz < f ≤ 10 Hz), alpha (8 Hz < f ≤ 12 Hz), and beta (12 Hz < f ≤ 30 Hz) bands are extracted from all 14 electrodes as features. In addition, the difference between the spectral power of all possible symmetrical pairs on the right and left hemisphere is extracted to measure the possible asymmetry in the brain activity due to the valance of emotional stimuli [41]. The asymmetry features were extracted from four symmetric pairs over four bands, AF3–AF4, F7–F8, F3–F4, and FC5–FC6. The total number of EEG features of a trial for 14 electrodes is 14 × 4 + 4 × 4 = 72.

## 3. Results

### 3.1. Preliminary Correlation Analysis

**Figure 9:**
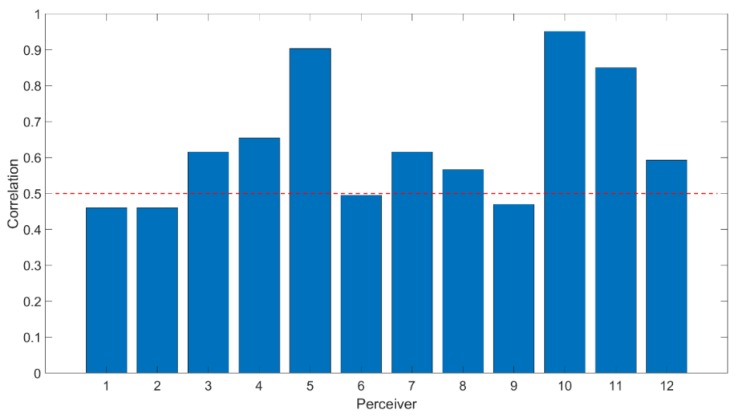
Correlation of the participants’ facial affective states and the ground truth over all trials. The average is 0.5 marked with the red dashed line.

In this study, the correlation was calculated using the ground truth for all video and image trials (the given affective labels on image stimuli and participants’ self-assessments on video stimuli) and the facial affective states measured by the automatic facial emotion recognition system. The self-assessment has been widely used to measure mental states in the literature [7,58]. The result in Figure 9 shows that all participants’ face affective states show a positive correlation with those reported by participants (*p* < 0.01). It complies with our hypothesis that the facial affective expression may reflect the affective state in the mind. However, it is likely that the self-assessment provided by the participant is derived from the participants’ recall or from second thoughts. In this section, we examine the relationship between the facial affective expressions and mental states. In order to support the hypothesis, we build the model for further analysis as detailed in this section.

Moreover, we looked at the correlation of brain activities with the affective states. Please see Appendix A Table A1 and Table A2. The findings indicate that lower frequency band signals are more highly correlated with positive affective states delivered by participants who are triggered by both video and images. The correlation from the frontal head is slightly higher than that in posterior of the head. The findings are in line with the previous work [66].

### 3.2. Affective State Detection from Brain Activity

There were forty trials (twenty images and twenty videos as mentioned in the section of Methods) for each participant. Polynomial Support Vector Machine (SVM) was used for classification. fNIRS and EEG features were concatenated to form a larger feature vector before feeding them into the model. For comparison, we also applied the univariate modality of either fNIRS or EEG features for recognition of the affective state. We applied a leave-one-out approach for training and testing. That is, the features of nineteen trials over all participants were extracted to train the model. The remaining one trial of each participant was used for testing. The performance of the experiment was validated through twenty-times iterations.

**Figure 10:**
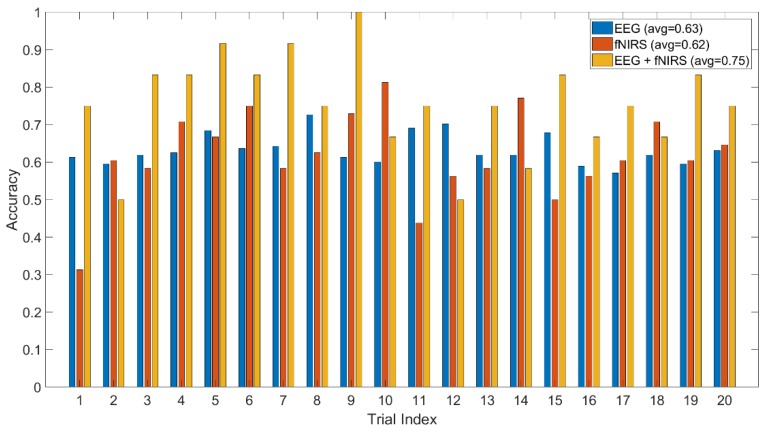
Comparison of the proposed method (EEG + fNIRS) and the ones where only EEG or fNIRS employed for all image-content trials.

**Figure 11:**
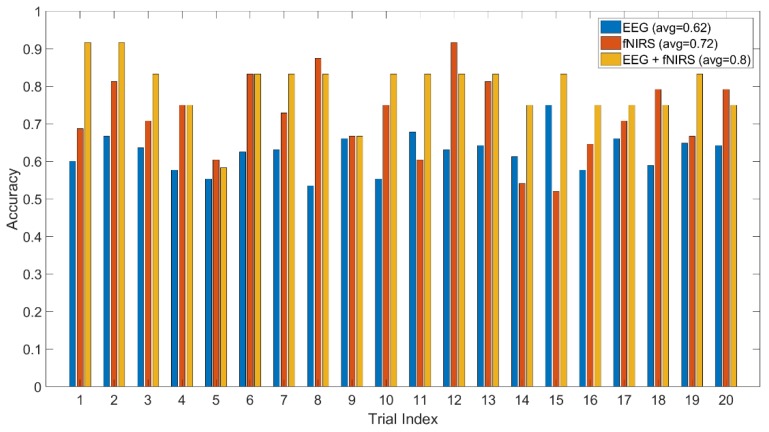
Comparison of the proposed method (EEG + fNIRS) and the ones where only EEG or fNIRS employed for all video-content trials.

**Figure 12:**
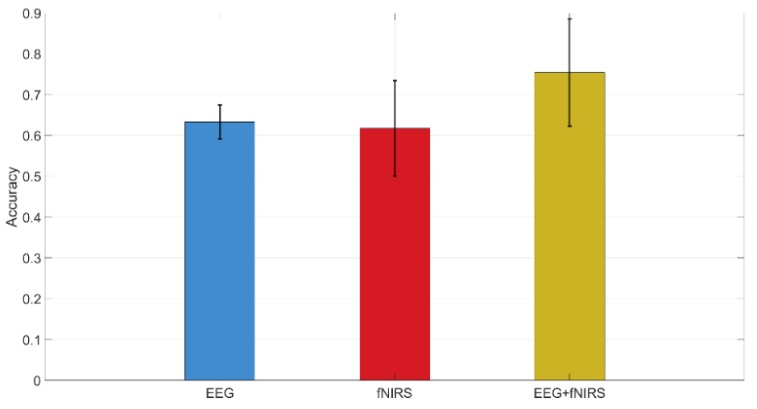
Performances of the proposed fNIRS + EEG method along with EEG only and fNIRS only techniques for all image-content trials as displayed. Whiskers are standard deviation.

**Figure 13:**
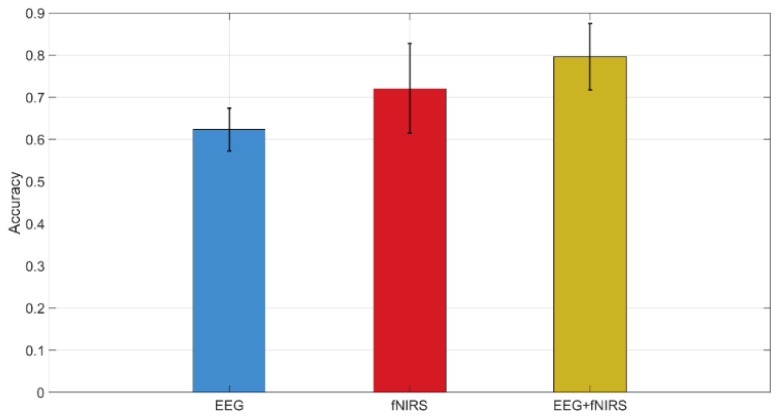
Performances of the proposed fNIRS + EEG method along with EEG only and fNIRS only techniques for all video-content trials as displayed. Whiskers are standard deviation.

**Figure 14:**
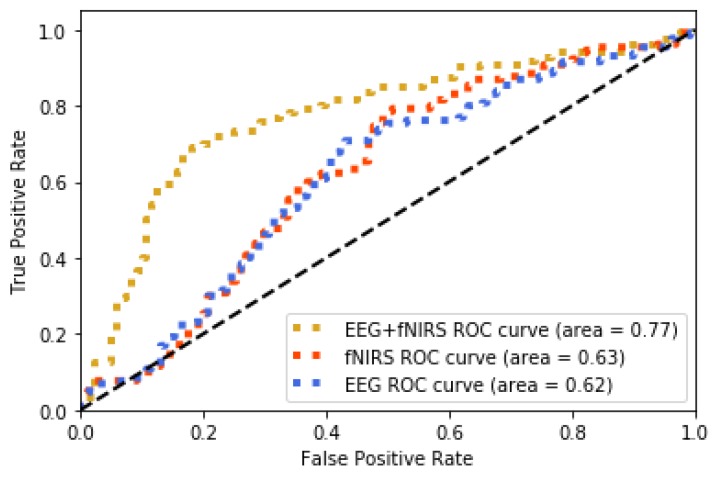
ROC curve comparison of the proposed method (EEG + fNIRS) and the ones where only EEG or fNIRS employed for all image-content trials

**Figure 15:**
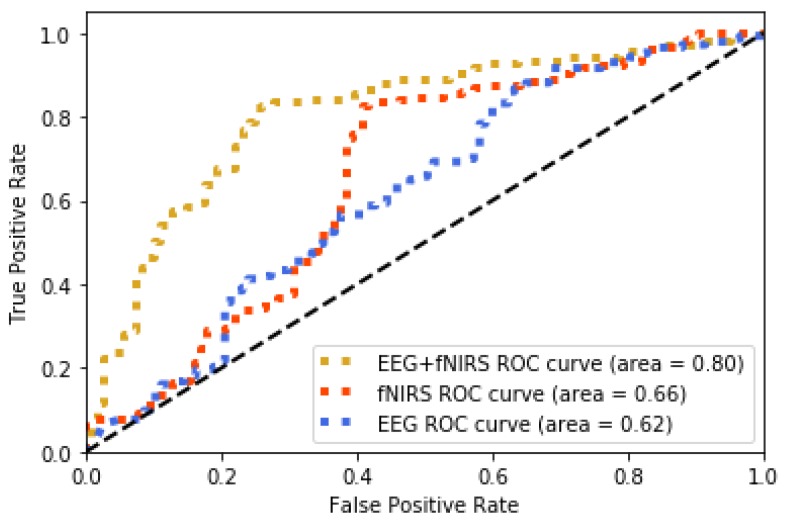
ROC curve comparison of the proposed method (EEG + fNIRS) and the ones where only EEG or fNIRS employed for all video-content trials.

The method jointly using fNIRS and EEG features shows 0.75 accuracy of recognition (image-content stimuli), which outperforms the techniques where only one of them is utilized (0.63 for EEG and 0.62 for fNIRS). The same finding is observed from the experiment using video-content stimuli (0.8 for EEG + fNIRS, 0.72 for fNIRS, and 0.62 for EEG). The recognition performance for joint use of fNIRS and EEG along with the cases where only one is used are displayed in Figure 10 and Figure 11 for image and video content type stimuli, respectively. This observation is consistent for almost all trials. In some trials, univariate modality of EEG or fNIRS shows higher performance. We observed that some participants reflected strongly to the stimuli while some participants showed higher affective tolerance to the same stimuli. This finding is consistent with our pilot study. The proposed multi-modal method performs over 10% better than the single-modality methods for the same stimuli. The average performances of these three methods are compared in Figure 12 and Figure 13 for image-content and video-content stimuli, respectively. The standard deviation error bars show the variability of the performance for all trials. The area of receiver operating characteristic (ROC) curve for EEG + fNIRS reaches 0.77 for image-content trials and 0.80 for video-content trials. The ROC curves in Figure 14 and Figure 15 also show that the performance for joint use of fNIRS and EEG exceeds the approach using only one of them. The standard error and 95% confidence intervals are calculated in Table 2 and Table 3. In addition, we found that the proposed method recognizes the affective response to video-content stimuli more accurately than those caused by image-content stimuli. It is likely that the dynamic (video) stimuli provide more contextual information than static (image) ones.

### 3.3. Similarity of Spontaneous Facial Affective States and Affective States Coded by the Brain Activity

To assess the spontaneous affective states estimated through brain activity, first we evaluate the reliability of the automatic facial emotion recognition system that is used to recognize the participants’ facial reaction to the given stimulus. In order to achieve this, we calculated the affect recognition accuracy of the system by comparing the detected results with the given labels on image stimuli and participants’ self-assessment on video stimuli. The affect recognition rate of the system for each trial are tabulated in Table 4 and Table 5 for image and video-content trials, respectively. *T_i_*, *i* = 1, …, 20 represents the *i*th trial. The overall accuracy of the system reaches 0.74 (σ = 0.10) for image-content stimuli and 0.80 (σ = 0.10) for video. The results are satisfactory and indicate that the system performs well to detect a person’s spontaneous facial expressions.

Next, we estimated the degree of the similarity of spontaneous affective states expressed on the face and those coded by brain activity (EEG + fNIRS). Similarity scores were calculated by the correlation of spontaneous facial affect recognized by the system and the affective states translated by participants’ brain signals across video-content and image-content trial per participant, respectively. The results in Table 6 and Table 7 show that the affective states expressed on face is correlated to that delivered through brain triggered by both video and image stimuli, which is significant at *p* < 0.05. That is, the spontaneous facial affective states can reflect the true brain affective responses to the stimuli.

## 4. Discussion

This study provides new insights for the exploration and analysis of spontaneous facial affective expression associated with simultaneous multimodal brain activity in the form of two wearable and portable neuroimaging techniques—fNIRS and EEG—that measure hemodynamic and electrophysiological changes, respectively. We have demonstrated that affective states can be estimated from human spontaneous facial expressions and brain activity via wearable sensors. The experimental results are founded on the premise that the participant has no knowledge of stimuli prior to the experiment. The spontaneous facial expressions of participants can be triggered by emotional stimuli. Moreover, specific neural activity changes are found due to the perception of the emotional stimuli. In addition, we found that video-content stimuli more readily induce the participants’ affective states than image-content stimuli. This can be explained as dynamic (video) stimulus provides more contextual information than a static (image) one. Compared to the static (image) stimuli, dynamic (video) ones trigger enhanced emotion delivered by brain activity as also shown in [67].

In this study, the findings were derived from the combined analysis of cortical hemodynamic and electrophysiological signals. The neural activities were measured by two non-invasive, wearable and complementary neuroimaging techniques, fNIRS and EEG. The complementary nature of fNIRS and EEG has been reported in the literature with multimodality studies [43,47,68,69,70]. Particularly, both of them have received considerable attention on emotion inference and emotional mapping on brain activities [21,41,71]. The proposed hybrid method for affective state detection jointly using fNIRS and EEG signals outperforms techniques that employ only EEG or only fNIRS. The same results are observed using both video or image-content types of stimuli. The method jointly using fNIRS and EEG features shows 0.8 accuracy (video-content stimuli) and 0.75 accuracy (image-content stimuli) which outperforms the techniques where only one of them is utilized. The results here confirm earlier multimodal fNIRS + EEG studies and highlight the complementary information content in both signal streams [47].

The video stream to measure facial reactions to different stimuli offers prompt, objective, and accurate recognition performance in continuous time. The regional facial features are highlighted since they convey significant information relevant to expressions. It is natural to classify the states of each facial region rather than considering the holistic features of the entire face for recognition [72]. The experimental results support our hypothesis by showing a high correlation between recognized facial affective expressions and the ground truth for all trials (the given labels on image stimuli and participant’s self-assessment on video stimuli).

The study described here provides important albeit preliminary information about wearable and ultra-portable neuroimaging sensors. It is important to highlight the fact that EPOC EEG electrodes are sensitive to external interference and non-brain signal sources such as muscle activity. Long, thick hair of participants could prevent electrodes from touching the scalp properly in order to collect “clean” brain signals. The challenging nature of measuring EEG signals may cause an adverse effect on our analysis of the relationship of facial activities and the affective states translated by their brain signals. Moreover, the fNIRS measures of the PFC hemodynamic response were used based on earlier studies [21]; however, monitoring of other brain areas could increase the overall classification accuracy. Finally, some prior work has shown that men and women differ in the neural mechanisms underlying their expression of specific emotions [73]. It is noted that all subjects involved in this study were male. However, future work may extend this study and its findings to all sexes.

The video sequences and images used in this study display short duration content, although all participants stated that they were able to understand all stimuli. However, it is of interest to address how the participants react to the content stimuli with longer durations in future studies. The findings in this study indicate that the spontaneous facial affective expressions are interrelated to the measured brain activity. It is likely that facial reactions to the longer duration-content stimuli might differ in frequency. The audience’s physiological responses to two-hour long movies were measured in [74] and revealed significant variations in affective states throughout the media. The extension of this work might benefit the specific applications that require the feedback of longer-duration content such as online education and entertainment. Also, the accuracy score per subject must be interpreted with caution. In a two class and ten testing trials per class to fit with experimental constraints, classification performance should be higher than 70% to be statistically significant (*p* < 0.05) [75,76]. Considering both image-content and video-content, average performance of classifier with EEG + fNIRS passed this limit. Further improvements with preprocessing methods and/or machine learning methodologies could improve and optimize the classifier performance.

## 5. Conclusions

To the best of our knowledge, this is the first attempt to detect affective states by jointly using fNIRS, EEG, and capture of facial expressions. The study reveals a strong correlation between spontaneous facial affective expressions and the affective states delivered by brain activities. The experimental results show that the proposed EEG + fNIRS multimodal method outperforms fNIRS-only and EEG-only approaches. The experimental results confirm the feasibility of the proposed method. In addition, the results highlight the reliability of spontaneous facial expression and use of wearable neuroimaging as promising methodologies to serve for various practical applications in the future. As the sensors used in the study allow untethered and mobile measurements, the approach demonstrated can be readily adapted in the future for measurements in real-world settings.

## Figures and Tables

**Table 1:** States of three face regions.

Face Regions		Observable States
Eyebrows		raise, fall, neutral
Eyes		open, close, neutral
Mouth	Mouth	open, close, neutral
	Lips corners	up, down, pull, pucker, neutral

**Table 2:** Performance comparisons of the proposed method (EEG + fNIRS) and the ones where only EEG or fNIRS employed for all image-content trials.

Model	Observation	ROC Area	Standard Error	95% Confidence Interval
EEG+fNIRS	240	0.77	0.02	0.74–0.80
fNIRS	240	0.63	0.03	0.57–0.69
EEG	240	0.62	0.02	0.58–0.66

**Table 3:** Performance comparison of the proposed method (EEG + fNIRS) and the ones where only EEG or fNIRS employed for all video-content trials.

Model	Observation	ROC Area	Standard Error	95% Confidence Interval
EEG+fNIRS	240	0.80	0.03	0.75–0.85
fNIRS	240	0.66	0.03	0.61–0.71
EEG	240	0.62	0.02	0.58–0.66

**Table 4:** Affect recognition rate of system for each image-content Trial.

**T1**	**T2**	**T3**	**T4**	**T5**	**T6**	**T7**	**T8**	**T9**	**T10**
0.92	0.75	0.67	0.75	0.83	0.75	0.67	0.83	0.83	0.83
**T11**	**T12**	**T13**	**T14**	**T15**	**T16**	**T17**	**T18**	**T19**	**T20**
0.67	0.75	0.67	0.5	0.75	0.75	0.92	0.75	0.67	0.58

**Table 5:** Affect recognition rate of system for each video-content Trial.

**T1**	**T2**	**T3**	**T4**	**T5**	**T6**	**T7**	**T8**	**T9**	**T10**
0.83	0.67	0.83	0.59	0.92	0.83	0.67	0.83	0.92	1
**T11**	**T12**	**T13**	**T14**	**T15**	**T16**	**T17**	**T18**	**T19**	**T20**
0.75	0.83	0.83	0.83	0.83	0.83	0.67	0.92	0.67	0.83

**Table 6:** Correlation of spontaneous facial affect and affect state translated by participants’ brain signals triggered by image stimuli.

	**Participant 1**	**Participant 2**	**Participant 3**	**Participant 4**	**Participant 5**	**Participant 6**
phi coefficient	0.58	0.45	0.50	0.54	0.49	0.47
*p* value	0.008	0.045	0.023	0.013	0.027	0.036
	**Participant 7**	**Participant 8**	**Participant 9**	**Participant 10**	**Participant 11**	**Participant 12**
phi coefficient	0.81	0.64	0.61	0.74	0.68	0.68
*p* value	0.000	0.002	0.004	0.000	0.001	0.001

**Table 7:** Correlation of spontaneous facial affect and affect state translated by participants’ brain signals triggered by video stimuli.

	**Participant 1**	**Participant 2**	**Participant 3**	**Participant 4**	**Participant 5**	**Participant 6**
phi coefficient	0.47	0.70	0.59	0.51	0.49	0.52
*p* value	0.036	0.001	0.006	0.020	0.027	0.018
	**Participant 7**	**Participant 8**	**Participant 9**	**Participant 10**	**Participant 11**	**Participant 12**
phi coefficient	0.81	0.58	0.52	0.55	0.50	0.68
*p* value	0.000	0.008	0.018	0.011	0.023	0.001

## Appendix A

To illustrate the affective states (positive or negative) in different brain regions, four frequency bands as Power Spectral Density (PSD) features shown in Table A1 and Table A2 extracted from EEG signals to identify brain patterns. The tables show the correlation between EEG spectral density in selected frequency bands and the spontaneous affective state conveyed from participants via 14 electrodes. The finding indicates that lower frequency band signals is more highly correlated with positive affective state delivered by participants who are triggered by both video and image. The correlation from frontal head is slightly higher than that in posterior of head. The findings are in line with the previous work [66].

**Table A1:** Correlation between EEG PSD frequency bands and ground truth after triggered by image contents where the significant correlation (*p* value ≤ 0.05) is indicated with *.

	Anterior					Posterior				Anterior				
	AF3	F7	F3	FC5	T7	P7	O1	O2	P8	T8	FC6	F4	F8	AF4
**Theta**	0.72	0.62	0.61	0.57	0.81	0.48	0.69	0.59	0.53	0.48	0.56	0.68	0.61	0.46
**Slow Alpha**	0.69	0.59	0.61	0.56	0.80	0.47	0.68	0.59	0.53	0.48	0.55	0.68	0.60	0.46
**Alpha**	0.11 *	0.10	0.55	0.48	0.67	0.38	0.51	0.56	0.51	0.42	0.53	0.66	0.58	0.45
**Beta**	−0.32	−0.24	−0.15	−0.32	−0.19	−0.21	−0.30	0.11 *	0.25	−0.13	0.10	0.24	0.20	0.25

* The correlation with *p* value >0.05.

**Table A2:** Correlation between EEG PSD frequency bands and ground truth after triggered by video contents where the significant correlation (*p* value ≤ 0.05) is indicated with *.

	Anterior					Posterior				Anterior				
	AF3	F7	F3	FC5	T7	P7	O1	O2	P8	T8	FC6	F4	F8	AF4
**Theta**	0.61	0.58	0.74	0.38	0.62	0.46	0.54	0.47	0.46	0.56	0.47	0.56	0.51	0.47
**Slow Alpha**	0.60	0.56	0.73	0.37	0.62	0.46	0.53	0.46	0.46	0.55	0.46	0.55	0.51	0.46
**Alpha**	0.50	0.19	0.64	0.33	0.54	0.41	0.43	0.36	0.44	0.50	0.16 *	0.47	0.46	0.41
**Beta**	−0.21 *	−0.22	−0.41	−0.08 *	−0.30	−0.09	−0.29	−0.23	0.18	−0.05	−0.54	−0.19	−0.09	−0.15

* The correlation with *p* value >0.05.
